# Unfractionated heparin as a safe alternative in a case of low molecular weight heparin-induced thrombocytosis: A case report

**DOI:** 10.1016/j.amsu.2021.102370

**Published:** 2021-04-30

**Authors:** Salem Jabira, Hassan Mitwally, Mohamed Saad, Edin Karic, Khaled Gazwi, Hani Elzeer, Moustafa Elshafei

**Affiliations:** a Department of Critical Care, Al-Wakra Hospital, Hamad Medical Corporation, Qatar; b Department of Pharmacy, Al-Wakra-Hospital, Hamad Medical Corporation, Qatar

**Keywords:** Thrombocytosis, Heparin, Low-molecular-weight, Enoxaparin, Drug-related side effect, Adverse reaction

## Abstract

**Introduction and importance:**

Low molecular weight heparins are rarely associated with thrombocytosis. However, the safety of transitioning to unfractionated heparin is unknown.

**Case presentation:**

We report a case of a 47-year-old South Asian male who presented to the hospital after ingestion of a caustic liquid. He received subcutaneous enoxaparin 40 mg once daily for prophylaxis against venous thromboembolism. His platelet count increased from the baseline of 748 × 10^9^/L to a peak of 1213 × 10^9^/L, after which enoxaparin was changed to unfractionated heparin. His platelet count returned to normal within seven days. The modified Naranjo scale with thrombocytosis-specific criteria was 6, indicating a probable association with enoxaparin.

**Clinical discussion:**

In this case, the patient developed thrombocytosis after initiation of low-molecular weight heparin and platelet count normalized after shifting to unfractionated heparin.

**Conclusion:**

Clinicians should suspect LMWH-induced thrombocytosis when platelet count elevation cannot be explained by other causes. Unfractionated heparin might be a safe alternative in case of low molecular weight heparin-induced thrombocytosis.

## Highlights

• Low molecular weight heparin-induced thrombocytosis is a rare adverse drug reaction.
• Clinicians should anticipate low molecular weight induced thrombocytosis when other causes of thrombocytosis are excluded.
• Unfractionated heparin may be a safe therapeutic alternative in case of low molecular weight induced thrombocytosis.

## 1 Introduction

Low molecular weight heparin (LMWH) is considered the drug of choice in preventing venous thromboembolism (VTE) in high risk surgical or medical hospitalized patients [1,2]. LMWH is associated with several adverse drug events such as bleeding, thrombocytopenia, and hypoaldosteronism [3,4]. LMWH is rarely associated with the incidence of thrombocytosis [5,6]. It is not known whether this adverse effect is related to the heparin moiety of LMWHs. Consequently, the safety of using unfractionated heparin in cases of LMWH-induced thrombocytosis is unclear. In this case report, we report a patient who developed enoxaparin-induced thrombocytosis which recovered after transitioning to unfractionated heparin.

### 1.1 Case

A forty-seven-year-old South Asian male patient with a previous history of hypertension, admitted to our hospital on the August 14, 2019 with severe epigastric pain for two days associated with bloody vomiting. On presentation; his blood pressure was 183/124 mmHg, heart rate was 93 bpm, respiratory rate was 22 bpm, and oxygen saturation was 93% on room air. His venous blood gas result showed pH 7.031, PCO_2_ 36 mmHg, PaO_2_ 50 mmHg, potassium 5.1 mEq/L, sodium 144 mEq/L and HCO_3_ 9.6 mEq/L. Blood counts on the day of admission were as follows: white blood cells 16.4 × 10^3^/μL, hemoglobin 13.2 g/dL, platelets 136 × 10^9^/L. The patient was intubated in the emergency department due to tachypnea, tachycardia, and severe metabolic acidosis; then he was shifted to the medical intensive care unit (MICU). An urgent computed tomography image of the abdomen was ordered, which revealed features of ischemic changes involving the wall of the stomach, duodenal, and proximal jejunal loops, plus features of acute pancreatitis. Laboratory investigation showed an increase in amylase and lipase, 508 and 974 U/L, respectively. Due to the suspicion of bowel ischemia, intravenous infusion of unfractionated heparin was started on the first day of admission with a target of therapeutic activated partial thromboplastin time. On the 3rd day of MICU admission, the patient was extubated successfully, and proper history was taken from him. The patient gave a history of ingesting an unknown liquid substance, after which he started to have these symptoms. On day five, the patient had hemoglobin drop, and esophago-gastro-duodenoscopy was done and showed diffuse esophageal, gastric, and duodenal wall changes suggestive of ischemic necrosis, suspecting caustic substance ingestion. A biopsy was taken, which showed totally necrotic tissues. The patient was started on parenteral nutrition and decided for conservative treatment.

**Fig. 1:**
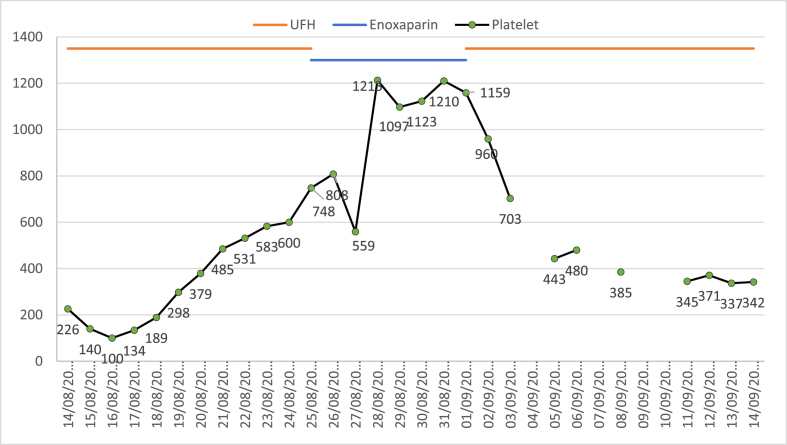
Trend of platelet counts with unfractionated heparin (UFH) and enoxaparin.

On day eleven of admission, the treating team decided that bowel ischemia is unlikely and changed the continuous intravenous infusion of unfractionated heparin to subcutaneous enoxaparin 40 mg once daily for venous thromboembolism (VTE) prophylaxis. Platelet count on the first day of enoxaparin was 748 × 10^9^/L, which increased to 1213 × 10^9^/L on day three of enoxaparin [Fig. 1]. All medications were reviewed for possible drug-induced thrombocytosis. Enoxaparin was stopped, and the patient was started on unfractionated heparin 5000 units subcutaneously every 12 hours as an alternative to enoxaparin for VTE prophylaxis. After two days from enoxaparin discontinuation, platelets dropped to 703 × 10^9^/L. On day seven of enoxaparin discontinuation, the platelet count dropped to 385 × 10^9^/L, and it reached 345 × 10^9^/L on day ten of enoxaparin discontinuation. During the time of platelet count elevation, the patient was afebrile and receiving no oxygen support. All cultures sent were negative, the C-reactive protein was only 55 mg/L (decreasing from 208 mg/L upon admission), procalcitonin was 0.58 ng/mL (decreasing from 23 ng/ml upon admission), and no evidence of active infection during that time. The patient was transferred from the intensive care unit to the medical floor on day 17 of hospital admission for a continuation of care.

## 2 Discussion

Drug-induced thrombocytopenia is considered one of the causes of secondary or reactive thrombocytosis [7]. Many drugs were reported previously to cause thrombocytosis e.g. penicillins, cephalosporins, clozapine, and others [[7], [8], [9], [10], [11]].

In this case report, we assessed the possibility of drug-induced thrombocytosis using the Modified Naranjo Scale with thrombocytosis-specific criteria [7]. The score was 6, which indicated probable causality, as shown in Table 1.

**Table 1:** Modified naranjo scale with thrombocytosis-specific criteria.

Criteria	Yes	No	Unknown	Score
More than four previous reports on this reaction	✓			+1
Documented platelet count of ≥500 × 10^9^/L	✓			+2
Temporally related resolution of thrombocytosis after withdrawal of the suspected drug	✓			+1
Reappearance of thrombocytosis temporally related to drug administration			✓	0
Patient has no infection, cancer, tissue damage, anemia, recent surgery, post-splenectomy, or chronic inflammatory process^a^		✓		−1
Did the reaction reappear when a placebo was given?^b^		✓		+1
Was the drug detected in the blood (or other fluids) in concentrations known to be toxic?			✓	0
Was the reaction more severe when the dose was increased, or less severe when the dose was decreased?			✓	0
Did the patient have a similar reaction to the same or similar drugs in any previous exposure?			✓	0
Was thrombocytosis confirmed by serial platelet counts 500 × 10^9^/L?	✓			+1
Score interpretation: 1–4 = Possible, 5–8 = Probable, ≥ 9 = definite.				6 Probable

a The patient was unlikely to have an infection but considered to have tissue damage and ongoing inflammatory process secondary to his acute pancreatitis and tissue necrosis.

b The patient received unfractionated heparin without reappearance of thrombocytosis.

LMWH induced thrombocytosis has been infrequently reported. Hommel et al. reported a case of 42 years old man admitted to the hospital with a closed head injury and developed thrombocytosis reaching 1005 × 10^9^/L on day eight of enoxaparin. After discontinuation of enoxaparin, platelet count returned to normal within six days. Enoxaparin was re-initiated again, and platelets count increased eight days later to a peak of 920 × 10^9^/L [12]. Tonbul et al. reported a case of thrombocytosis in a 35-week newborn girl. Platelets count increased from 320 to 954 × 10^9^/L after ten days from starting enoxaparin 1.5 mg/kg every 12 hours to treat bilateral renal venous thrombosis. In this case, the patient was shifted to another LMWH, and platelets count normalized by day five from enoxaparin discontinuation (312 × 10^9^/L) [13].

French pharmacovigilance database reported 143 cases of thrombocytosis. Fifty-one patients treated with LMWHs had platelet counts >500 × 10^9^/L. The calculated relative reporting ratio is 27.5 (p < 0.0001; 95% CI 19.5 to 38.9). Agents suspected were; enoxaparin in 23 patients, nadroparin in 17 patients, dalteparin in 7 patients, and reviparin in 4 patients. All patients were asymptomatic [5]. In a retrospective study addressing the adverse drug reactions of LMWH, thrombocytosis was associated with enoxaparin (587 ± 102 × 10^9^/L) in 29 out of 95 patients [14]. However, the mean increase in platelet count was mild and may not be clinically important. LMWH induced thrombocytosis does not appear to be dose-related. Our patient developed the reaction with enoxaparin 40 mg daily, which is considered the smallest VTE prophylaxis dose in average weight adult without severe kidney functions (creatinine clearance > 30 ml/min). This dose was the same dose reported by Hommel et al. [12].

The mechanism of LMWH induced thrombocytosis thought to be secondary to megakaryocytes (MK) promotion. Fraxiparin found to potentiate in vitro stimulation of aplastic anemia serum, interleukin‐3, granulocyte‐macrophage colony‐stimulating factor, and erythropoietin on MK colony growth in vitro. Additionally, fraxiparin acted synergistically with heparin cofactor II and antithrombin III to promote megakaryocyte colony formation when compared to unfractionated heparin [15].

## 3 Conclusion

Clinicians should suspect LMWH-induced thrombocytosis when it cannot be explained by other causes. This adverse reaction is unlikely to be related to the heparin moiety. Unfractionated heparin might be a safe alternative in case of low molecular weight heparin-induced thrombocytosis.

This manuscript was written in line with the SCARE 2020 Guideline [16].

## Ethics approval

This study was approved by the ethical committee, Medical research center, Hamad Medical Corporation, under number: MRC-04-20-1101.

## Availability of data and materials

The datasets supporting the conclusions of this article are included within the manuscript.

## Authors' contributions

Salem Jabira: the conception and design of the study, acquisition of data, drafting the article, and approved the final manuscript.

Hassan Mitwally: acquisition of data, drafting the article and approved the final manuscript.

Mohamed Saad: acquisition of data, drafting the article, and approved the final manuscript.

Edin Karic: the conception and design of the study and approved the final manuscript.

Khaled Gazwi: the conception and design of the study and approved the final manuscript.

Hani Elzeer: drafting the article and approved the final manuscript.

Moustafa Elshafei: drafting the article and approved the final manuscript.

## Consent

Informed consent for publication of this case was obtained by the patient's next of kin.

## Registration of research studies

Not applicable.

## Guarantor

Salem Jabira.

Email: sjabira@hamad.qa

## Funding

This case report has no funds.

## Provenance and peer review

Not commissioned, externally peer-reviewed.

## Declaration of competing interest

All authors have no conflict of interest to declare.
